# Cobalt-Alloy Implant Debris Induce HIF-1α Hypoxia Associated Responses: A Mechanism for Metal-Specific Orthopedic Implant Failure

**DOI:** 10.1371/journal.pone.0067127

**Published:** 2013-06-20

**Authors:** Lauryn Samelko, Marco S. Caicedo, Seung-Jae Lim, Craig Della-Valle, Joshua Jacobs, Nadim J. Hallab

**Affiliations:** 1 Department of Orthopedic Surgery, Rush University Medical Center, Chicago, Illinois, United States of America; 2 Department of Immunology, Rush University Medical Center, Chicago, Illinois, United States of America; 3 Department of Orthopedic Surgery, Samsung Medical Center, Seoul, Korea; National Institutes of Health, United States of America

## Abstract

The historical success of orthopedic implants has been recently tempered by unexpected pathologies and early failures of some types of Cobalt-Chromium-Molybdenum alloy containing artificial hip implants. Hypoxia-associated responses to Cobalt-alloy metal debris were suspected as mediating this untoward reactivity at least in part. Hypoxia Inducible Factor-1α is a major transcription factor involved in hypoxia, and is a potent coping mechanism for cells to rapidly respond to changing metabolic demands. We measured signature hypoxia associated responses (i.e. HIF-1α, VEGF and TNF-α) to Cobalt-alloy implant debris both in vitro (using a human THP-1 macrophage cell line and primary human monocytes/macrophages) and in vivo. HIF-1α in peri-implant tissues of failed metal-on-metal implants were compared to similar tissues from people with metal-on-polymer hip arthroplasties, immunohistochemically. Increasing concentrations of cobalt ions significantly up-regulated HIF-1α with a maximal response at 0.3 mM. Cobalt-alloy particles (1 um-diameter, 10 particles/cell) induced significantly elevated HIF-1α, VEGF, TNF-α and ROS expression in human primary macrophages whereas Titanium-alloy particles did not. Elevated expression of HIF-1α was found in peri-implant tissues and synovial fluid of people with failing Metal-on-Metal hips (n = 5) compared to failed Metal-on-Polymer articulating hip arthroplasties (n = 10). This evidence suggests that Cobalt-alloy, more than other metal implant debris (e.g. Titanium alloy), can elicit hypoxia-like responses that if unchecked can lead to unusual peri-implant pathologies, such as lymphocyte infiltration, necrosis and excessive fibrous tissue growths.

## Introduction

The well established ability of total joint implants to restore function and mobility has been recently shocked by the unexpected early failures of some artificial hip implants, (up to 5% failure as early as 6 years post-operatively of some metal on metal designs) [1]–[3]. These failures are attributed to excessive exposure to metal debris, e.g. Cobalt-Chromium-Molybdenum-alloy (Co-alloy) metal debris. The pathophysiological reasons for this remain largely unknown. But they are different than the well established long term (>15 years), mechanisms by which particulate plastic and metal debris cause failure by inducing a subtle but persistent innate macrophage inflammatory responses (i.e. granulomas) that invades the bone-implant interface and leads to bone resorption and eventual aseptic loosening of the implant [4]–[7].

Sites of inflammation or tissue damage are generally characterized by decreased availability of oxygen, leading to a hypoxic state, which in turn further promotes inflammation and tissue damage at the affected area [8]. Myeloid cells (e.g. macrophages) of innate immunity have the capability to respond and function in a hypoxic (or hypoxic-like) microenvironment in order to maintain viability, activity and tissue homeostasis [9]. Macrophages are key mediators of inflammatory responses in hypoxic tissues and it has been proposed that a state of hypoxia can act as an “inflammatogen” and activate macrophages that infiltrate hypoxic tissues [8], [10], [11]. Hypoxia inducible factors (HIFs) 1 and 2 are known as primary transcription factors involved in the adaptation of cells to a hypoxic and inflammatory state [9]. HIF-1 is a major transcription factor activated in hypoxia, and is a potent coping mechanism for cells that once activated results in the ability for cells to rapidly respond to changing metabolic demands [8]. HIF-1 is a heterodimeric protein that is formed by two subunits: HIF-1α and HIF-1β. The subunit HIF-1β is constitutively expressed while HIF-1α is O_2_ sensitive and is closely associated with myeloid cells inflammatory response and microbicidal activities [8], [12]. In a hypoxic state, HIF-1α subunit stabilizes, translocates to the nucleus and dimerizes with HIF-1β beginning the transcription of hypoxia inducible genes, such as vascular endothelial growth factor (VEGF), a potent angiogenic factor [13]. A hypoxic microenvironment can also contribute to a highly inflamed state by increasing the secretion of pro-inflammatory cytokines [10]. In the context of orthopedics, recent studies have linked VEGF to the development of aseptic loosening and bone resorption [14], [15]. In this study we examined the expression of HIF-1α protein, a signature hypoxia-related transcription factor upstream of VEGF [13]. We investigated if HIF-1α expression contributes to metal-implant debris induced aseptic inflammation, particularly in cases of elevated exposure to metal implant debris (e.g. in metal-on-metal, MoM hip replacement implants). Both *in vitro* and *in vivo*, we examined whether MoM arthroplasty debris (Cobalt-alloy) preferentially to other metals (e.g. Titanium alloy) induces local pathology responses by creating cobalt-induced hypoxic-like responses as evidenced by the production and accumulation of HIF-1α protein and known associated reactions (i.e. inflammatory and angiogenic).

## Materials and Methods

### Ethics Statement

This study and consent process received approval from the Rush University Institutional Review Board (10052606-IRB01-CR02). Participants provided their written consent to participate in this study, and participation was limited to a one time blood draw or banked tissue specimens. All participants were 18 years of age or older.

### Media and Challenge Agents

Growth medium for the human monocyte cell line THP-1 (ATCC) was RPMI 1640 supplemented with L-Glutamine, Penicillin, Streptomycin, 25 mM Hepes (Lonza, Walkersville, MD USA) and 10% heat inactivated fetal bovine serum (FBS; Hyclone Laboratories, Logan, UT). Human primary monocytes were cultured in RPMI 1640 supplemented with L-Glutamine, Penicillin, Streptomycin, 25 mM Hepes and 10% heat inactivated autologous serum.

The mean particle sizes of Cobalt-alloy (Co-alloy, approx 60%Co, 28%Cr, <6%Molybdenum, <1%Nickel, ASTM F75) and Titanium-alloy (Ti-alloy, 90%Ti, 6%Al, 4%V, ASTM F136) particles were characterized by using laser diffraction and Scanning Electron Microscopy. Particles have a mean diameter of 1.2 µm (>95% <3 um) for Ti alloy and 0.9 µm (>95%<3 um) for Co-alloy particles. Subsequent to characterization, particles were cleaned, sterilized, and tested for endotoxin levels before use in experiments (<0.01 eU, Kinetic QCL, Bioengineering Solutions Inc, Oak Park, IL). Cobalt ions were produced using CoCl_2_ (a known hypoxic mimetic) at a final concentration of 0.1 mM working solution in sterile H_2_O (Sigma-Aldrich, St. Louis, MO). Challenge concentrations were previously determined to be non-toxic after 24 hours challenge [6], [16], [17].

### Monocyte/macrophage Cell Line

Human THP-1 monocytes were differentiated into macrophages by culturing 2.0×10^5^ monocytes in 48 well plates or 1.0×10^5^ in 96 well plates with phorbol ester (TPA) for 18–24 hours prior to metal challenge. Newly differentiated macrophages were challenged with particulate and soluble metals for 6 hours or 16 hours at 37°C 5% CO_2_ and supernatants were subsequently analyzed for mature Il-1β, VEGF, and TNF-α production.

### Primary Monocyte/Macrophage Cells

Peripheral blood monocytes were obtained from healthy donors (n = 4) by venipuncture with IRB-approved informed consent. Peripheral blood mononuclear cells (PBMCs) were isolated by Ficol gradient separation and collected for further purification. Peripheral blood CD14^+^ monocytes were isolated from PBMCs by negative selection with a specific magnetic bead antibody cocktail to CD3, CD7, CD16, CD19, CD56, CD123 and Glycophorin A (Miltenyi Biotec). Isolated human primary monocytes were assessed for >90% purity using FACS. Freshly isolated human primary macrophages were challenged with: Ti alloy particles of 1.2 µm at 1∶1, 5∶1, 10∶1, and 20∶1(particles∶cell) ratio, Co- alloy particles of 0.9 µm at 1∶1, 5∶1, 10∶1, and 20∶1(particles∶cell) ratio, and with 0.001 mM, 0.01 mM, 0.1 mM, and 0.3 mM CoCl_2_.

### Cytokine Analysis

Sandwich ELISAs for VEGF (R&D Systems) were used to detect metal-induced THP-1 and human primary macrophages VEGF secretion following manufacturer's instructions. Supernatants from particle challenged macrophages were collected at 6 hours and 16 hours of challenge for VEGF and stored at −80°C prior to analysis.

### Total HIF-1α Assay

Cell Based ELISA for HIF-1α (R&D Systems) was used to detect metal induced THP-1 and human primary macrophage total HIF-1α secretion in the context of the whole cell following the manufacturer's instructions. After fixation of the cells in the wells, the plate was read using a fluorescence plate reader with excitation at 540 nm and emission at 600 nm and a second reading was performed with excitation at 360 nm and emission at 450 nm. The total amount of HIF-1α in the cells is represented by the readings at 600 nm and the readings at 450 nm represented the amount of total Cytochrome C (housekeeping protein) in the cells. To determine the total amount of HIF-1α activated in the cells, the total HIF-1α fluorescence at 600 nm in each well was normalized to that of the total Cytochrome C fluorescence at 450 nm. The normalized triplicate readings for each sample are then averaged. The normalized averages of challenged cells were normalized to unchallenged control cells and represented as the percentage increase of HIF-1α expression in comparison to unchallenged control cells.

### Detection of HIF-1α in Human Tissue and Synovial Fluid Samples

Human/Mouse Total HIF-1α DuoSet intracellular ELISA (R&D Systems) was used to measure HIF-1α in homogenized peri-implant tissue samples from patients with MoM (n = 5) or MoP (n = 10) implants and in synovial fluid samples from patients with MoM (n = 4) or MoP (n = 5) implants following the manufacturer's instructions.

### Collection of Human Tissue Samples

Periprosthetic tissues were obtained from 5 patients with Metal-on-Metal (MoM) implant and from 10 patients with Metal-on-Polyethylene (MoP) implant undergoing primary revision surgery. All samples were freshly frozen in liquid nitrogen and stored at −80°C until time of analysis. Synovial fluid samples were also obtained from 4 patients with MoM implants and from 5 patients with MoP implants at the time of primary revision surgery. All synovial fluid samples were stored at −80°C

### Analysis of Human Tissue Samples

Periprosthetic tissue sample sections from patients with MoM (n = 5) and MoP (n = 10) implants and synovial fluid samples from MoM (n = 4) and MoP (n = 5) patients were treated with protease inhibitor (Sigma-Aldrich) following manufacturer's instructions prior to analysis and tissue samples were homogenized and centrifuged at 2000 rpm for 10 min in a microcentrifuge. Supernatants containing cellular proteins were subsequently collected and stored at −80°C. HIF-1α protein concentrations in the supernatants of periprosthetic tissue and synovial fluid samples were quantified using Human Mouse Total HIF-1α ELISA.

### Immunohistochemistry

Peri-implant tissues were used to detect the presence of HIF-1α using standard immunohistochemistry techniques. Briefly, the specimens were trimmed and excess bone fragments were removed to produce a 2 cm^2^ piece of tissue. Tissues were then placed in cassettes and covered with OCT embedding compound, placed in cooled isopentane, sectioned with a cryostat, fixed in anhydrous acetone for 20 seconds and stored at −70° C. The endogenous peroxides within the samples were blocked with peroxide (H_2_0_2_). Using primary and secondary antibodies combined with the Avitan-Biotin Complex (ABC)-horseradish peroxidase technique, macrophage intracellular and membrane bound cytokines were stained for HIF-1α.

### Cytokine Analysis

Supernatants from particle challenged THP-1 and human primary macrophages were collected after 6 hours and 16 hours of challenge for VEGF and TNF-α by ELISA (R&D Systems) following manufacturer's instructions.

### Reactive Oxygen Species (ROS) Assay

Intracellular generation of ROS was determined using oxidation of 2′,7′-dichlorodihydrofluorescein diacetate (H2DCFDA; Invitrogen). All THP-1 cells were plated at 1.5×105 cells per well in a black-clear bottomed 96-well plate (BD Falcon) in RPMI with 10% fetal bovine serum (FBS) with phorbol ester (TPA) for 18–24 h. Differentiated macrophages were challenged for 2.5 hours with: Ti alloy particles and Co-alloy particles at a ratio of 5∶1 (particles∶cell) ratio, 0.001 mM, 0.01 mM, and 0.1 mM CoCl2 (Cobalt ions). Culture medium was removed and followed by two washes 1X phosphate-buffered saline (PBS) to remove unbound particulate and soluble metals. The cells were then incubated with ROS fluorophore H2DCFDA at 10 µM for 30 min followed by two washes of 1X PBS. Fluorescence was measured (485 nm detection and 520 nm emission wavelengths). The fluorescence intensities of challenged cells were normalized to untreated controls for comparison.

### Statistical Analysis

One-way ANOVA with Bonferroni post test was used to determine significance for intragroup comparison of metal-treated macrophage cell line and human primary macrophages, where p value <0.05 was considered significant. Student's t-test was used for cytokine comparison to associated untreated controls.

## Results

**Figure 1:**
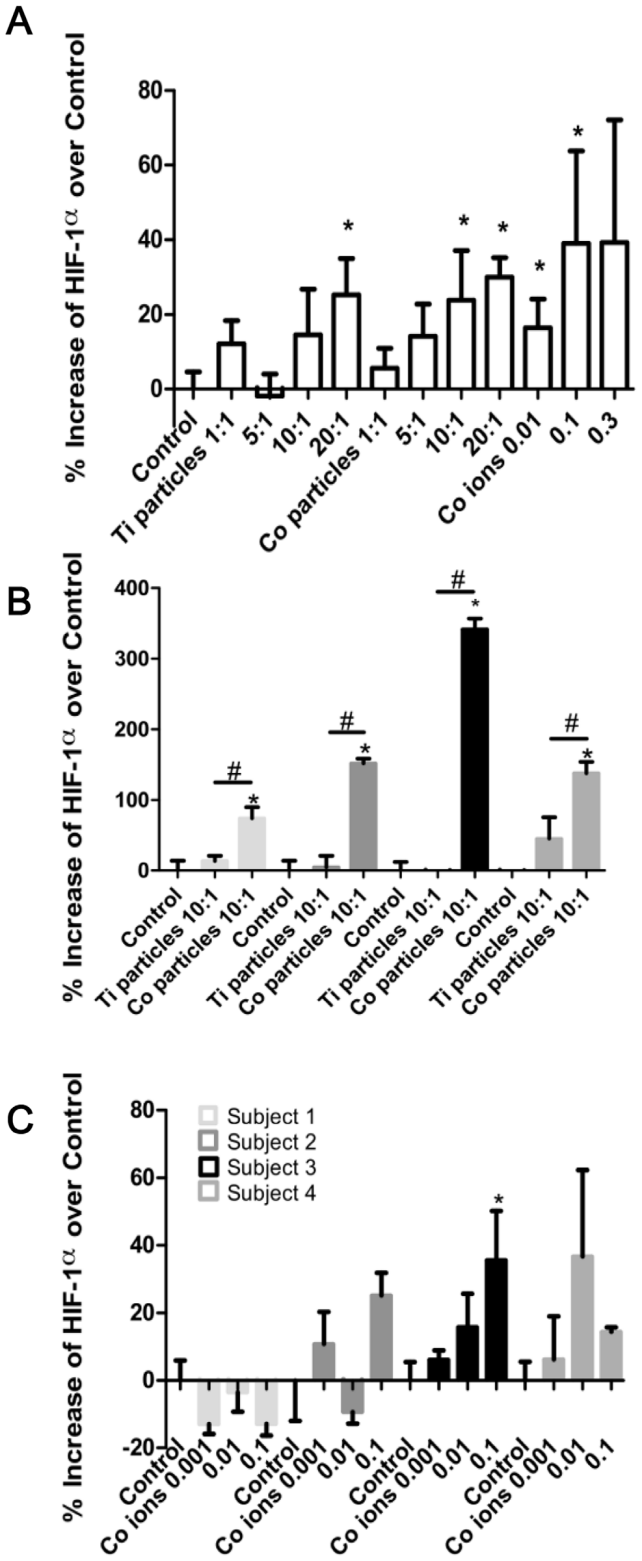
Total HIF-1α protein expression *in vitro*. (**A**) THP-1 macrophages (80,000cells/well) were cultured with increasing ratios of Ti-alloy and Co-alloy particles to cells (from 1/1, 5/1, 10/1, 20/1 particles/cell) or to increasing concentrations of CoCl_2_ for 6 hrs (0.01–0.3 mM). HIF-1α protein levels were determined using Total HIF-1α Cell-Based ELISA kit. Averages of challenged cells were normalized to unchallenged control cells and represented as the percentage increase of HIF-1α expression in comparison to unchallenged control cells. (**B–C**) Human primary macrophages (n = 4) were cultured with Ti-alloy and Co-alloy particles at a 10∶1 (particles∶cell) ratio for 6 hours (**B**) and with increasing concentrations of CoCl_2_ for 16 hours at 0.001–0.1 mM CoCl_2_ (**C**). HIF-1α protein levels were determined using Total HIF-1α Cell-Based ELISA kit. Averages of challenged cells were normalized to unchallenged control cells and represented as the percentage increase of HIF-1α expression in comparison to unchallenged control cells. Note: *p<0.05, determined by Student's t-test, # *p<0.05* ANOVA.

To determine if particulate and soluble metal implant debris induce a hypoxic (or hypoxic-like) micro-environment and activate the key transcription factor HIF-1α, we evaluated the response of differentiated THP-1 macrophages with increasing concentrations of Titanium-alloy (Ti-alloy) particles, Co-alloy particles and Cobalt-chloride ions, and measured THP-1 up-regulation of HIF-1α protein expression at 6 hours challenge (**Fig. 1A**). Increasing doses of Ti-alloy, Co-alloy particles and CoCl_2_ induced increased expression of HIF-1α in THP-1 macrophages compared to untreated controls. Moreover, increasing concentrations of cobalt ions significantly up-regulated HIF-1α with a peak response at 0.3 mM, likely due to other toxic effects of cobalt at concentrations higher than 0.3 mM. This type of reactivity dissipated by 16 hours in primary human macrophages indicating that HIF-1α protein half-life is likely relatively short (**Fig. 1C**). Primary human macrophages (n = 4 subjects) demonstrated comparable results to THP-1 macrophages when treated with Co-alloy particles at a ratio of 10∶1 (particles: cell). However, Ti-alloy particles only modestly induced HIF-1α protein expression in human primary macrophages (**Fig 1B**), significantly less than Co-alloy particles. Therefore, this *in vitro* data demonstrates that particulate Co-alloy debris derived from orthopedic implants can induce the stabilization of HIF-1α expression and suggests that Co-alloy particles are likely more bioreactive than Ti-alloy particles due to significant expression of HIF-1α in human macrophages.

**Figure 2:**
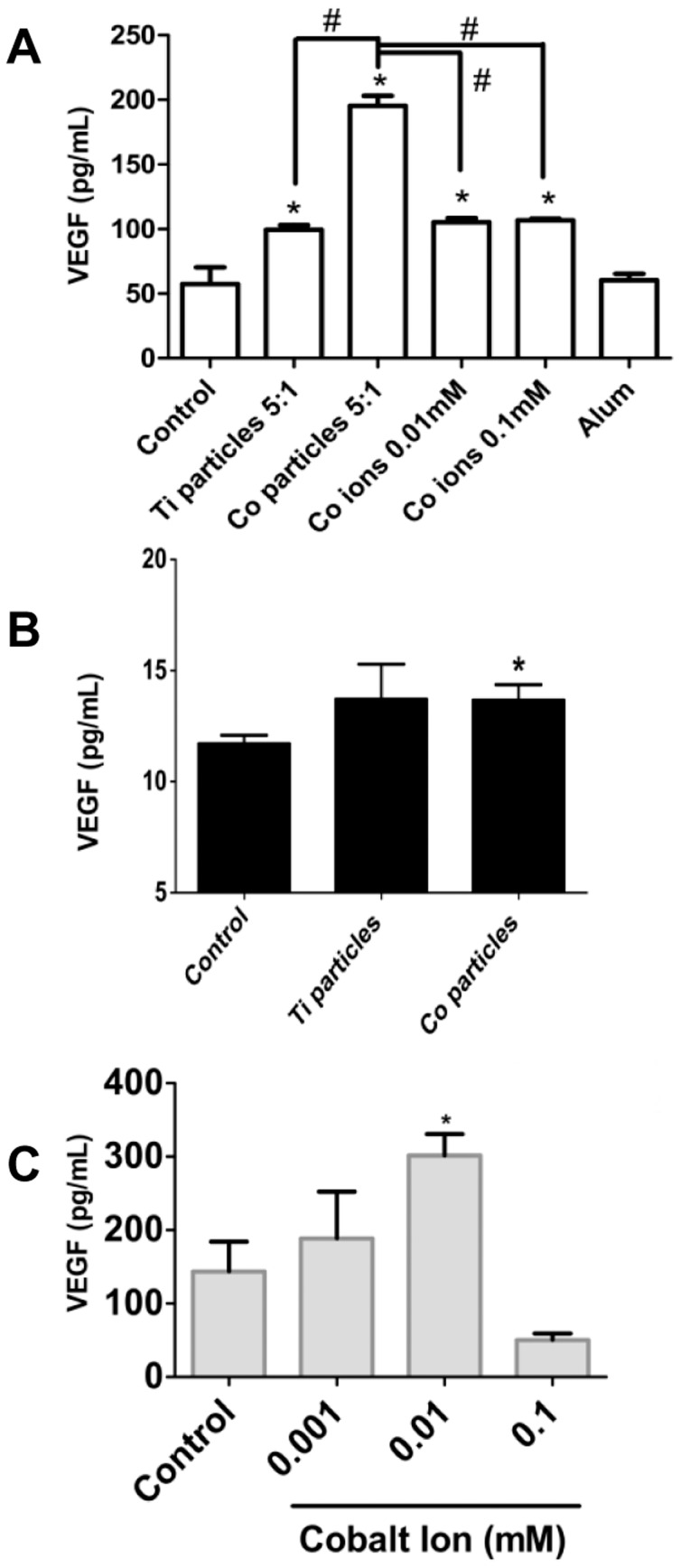
VEGF secretion due to particulate and soluble implant debris. (**A**) VEGF production was assessed after THP-1 differentiated macrophages were challenged with Ti-alloy (1.2 µm) and Co-alloy (0.9 µm) particles at 5∶1 (particles∶cell) ratio and with 0.01 mM and 0.1 mM CoCl_2_ for 16 hours and was quantified by ELISA. (**B**) One of four healthy human subjects (without an implant) demonstrated increased macrophage VEGF response to particle challenge with Ti-alloy and Co-alloy particles at 10∶1 ratio (particles∶cell), ELISA as early as 6 hrs. (**C**) One of n = 4 healthy human subjects (without an implant) demonstrated increased macrophage VEGF response to challenge with 0.001 mM, 0.01 mM, and 0.1 mM CoCl_2_ for 16 h. Culture supernatants were analyzed for VEGF by ELISA. Note: * p<0.05, Student's t-test compared to respective untreated controls, # p<0.05 ANOVA.

HIF-1α protein is a major transcription factor central in regulating hypoxia mediated gene expression [18] and the regulation of angiogenesis by HIF-1α is an important homeostatic mechanism to increase oxygen supply to meet metabolic demand [19]. Therefore, if HIF-1α was indeed up-regulated as a result of metal implant debris challenge, then downstream secretion of VEGF would also occur (**Fig 2A**). Thus as expected, Ti-alloy, Co-alloy particles and Co ions induced significant increases in VEGF secretion at 16 hours in the human macrophage cell line (THP-1). In proportion to HIF-1α, Co-alloy particles significantly increased VEGF by more than double that of Ti-alloy particles and cobalt ions. However, VEGF responses in primary human macrophages (n = 4) were not consistent in response to particle and ion challenge, compared to THP-1 macrophages, where only 2 of 4 subjects demonstrated a significant VEGF increase to either particles or ion Cobalt-based metal challenge as early as 6 hrs (Co-alloy particles at 6 hrs, **Fig. 2B**) and that this response increases over time (CoCl_2_ at 16 hrs, **Fig. 2C**). These data show that particulate and soluble Co-alloy implant debris can induce the secretion of VEGF in THP-1 macrophages in response to MoM implant debris due to HIF-1α upregulation; however, VEGF secretion by primary human macrophages appears to be more subject dependent, which may explain why metal-on-metal hip implant pathologies (e.g. pseudotumor formations) are not clinically found in all patients with MoM hip replacements with elevated levels of Co-alloy metal debris.

**Figure 3:**
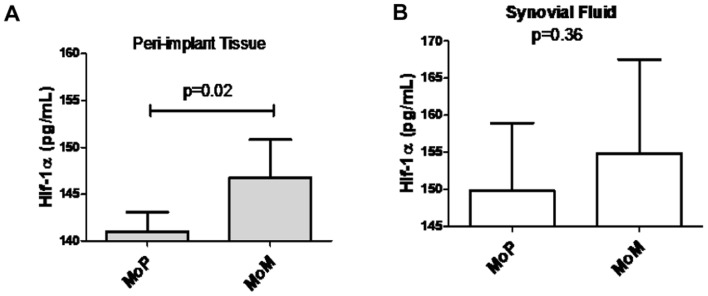
Total HIF-1α protein expression *in vivo*. (**A**) The expression level of total HIF-1α protein in peri-implant patient homogenized tissue samples of metal-on-metal (MoM) (n = 5) and metal-on-polythetheylene (MoP) (n = 10) implants and quantified with the Human/Mouse Total HIF-1α DuoSet IC ELISA. (**B**) The expression level of total HIF-1α protein in synovial fluid patient samples of metal-on-metal (MoM) (n = 4) and metal-on-polythetheylene (MoP) (n = 5) implants and quantified with the Human/Mouse Total HIF-1α DuoSet IC ELISA. Note: * p<0.05 determined by Student's t-test.

**Figure 4:**
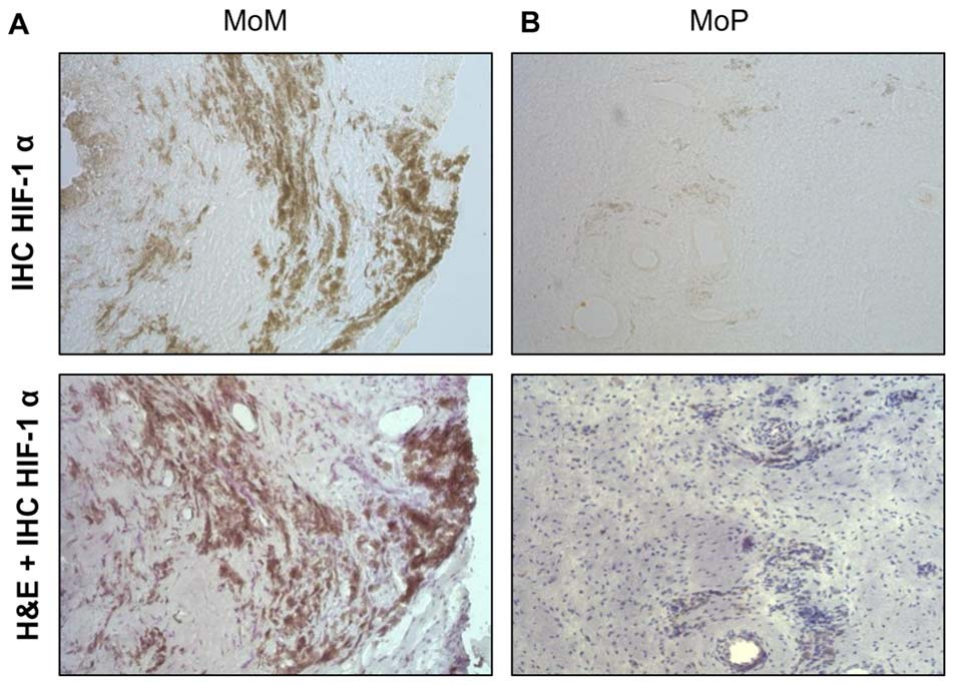
Immunohistochemistry analysis of HIF-1α protein expression in total hip replacements. (**A**) H&E staining and immunohistochemistry analysis of HIF-1α in peri-implant human tissue samples of a representative of n = 5 metal-on-metal hip replacements and (**B**) a representative of n = 10 metal-on-polymer hip replacements. All other tissues demonstrated similar patterns. All were revised for aseptic bone loss and pain where evidence of mild pseudotumor was present in all metal-on-metal tissues (original magnification 400x).

To determine if hypoxia induced by implant metals could be identified clinically *in vivo*, peri-implant tissues and hip joint synovial fluids of people with failing MoM total hip replacements were evaluated for HIF-1α expression using tissue homogenates and immunohistochemical analysis. We quantitatively analyzed HIF-1α protein expression in revision sample tissue sections of MoM and metal-on-polyethylene (MoP) implants to determine the differential expression of HIF-1α in implants that have an abundance of metal vs. polymer implant debris. We detected a significantly higher mean expression of HIF-1α in patients with failing MoM hips (n = 5) compared to MoP (n = 10) patient tissue samples (**Fig 3A**). This was supported by analysis of increased HIF-1α expression observed in the synovial fluid samples from MoM patients (n = 4) as compared to MoP (n = 5) patients (**Fig 3B**). To further characterize HIF-1α expression, we evaluated if HIF-1α protein was differentially present in MoM and MoP peri-implant tissues (**Fig. 4**). Immunohistochemistry analyses of HIF-1α expression was detected in both MoM and MoP peri-implant tissue samples; however, MoM peri-implant tissue samples expressed increased HIF-1α protein expression as compared to MoP tissue samples.

**Figure 5:**
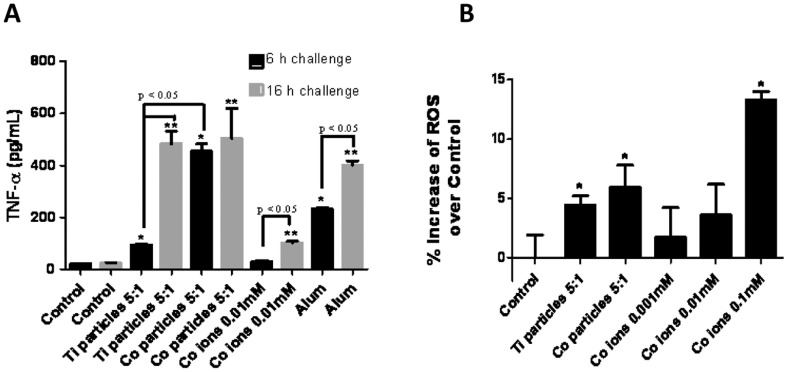
TNF-α and ROS production levels correlate with HIF-1α protein expression. (**A**) TNF-α cytokine production was assessed after THP-1 differentiated macrophages were challenged with Ti-alloy (1.2 µm) and Co-(0.9 µm) particles at a 5∶1 (particles∶cell) ratio and with 0.01 mM CoCl_2_ for 6 hours and 16 hours and was quantified by ELISA. (**B**) Intracellular generation of ROS was determined using oxidation of 2′,7′-dichlorodihydrofluorescein diacetate (H2DCFDA; Invitrogen). THP-1 differentiated macrophages were challenged with Ti-alloy (1.2 µm) and Co-(0.9 µm) alloy particles at 5∶1 (particles∶cell) ratio or with 0.001 mM, 0.01 mM, 0.1 mM CoCl_2_ for 2.5 h. The normalized averages of challenged cells are represented as the percentage increase of ROS production compared to unchallenged control cells. Note: *p<0.05, determined by Student's t-test, # *p<0.05* ANOVA.

Ti-alloy, Co-alloy and Cobalt ion challenged THP-1 macrophages (5∶1 ratio of particles per cell at 6 hours and 16 hours) induced significantly elevated TNF-α production (**Fig 5A**). And Co-alloy particles induced significant increase in TNF-α production when compared to Ti-alloy particles at 6 hours of challenge (p<0.05). These combined results demonstrate that different metals behave differently where preferentially Co-alloy particles induce a hypoxic or hypoxic-like micro-environment that results in HIF-1α accumulation that in turn augments NFκB signaling pathway and the downstream release of TNF-α in macrophages.

To determine if a possible mechanism by which particulate and soluble implant debris can result in the induction of HIF-1α activity, we evaluated intracellular production of ROS in THP-1 macrophages in response to Ti-alloy, Co-alloy particles and CoCl2 for 2.5 hours (**Fig. 5B**). Ti-alloy, Co-alloy particles and Co ions significantly increased ROS production in THP-1 cells compared to untreated controls (p<0.05). Moreover, ROS production correlates with HIF-1α protein expression in response to metal implant debris, with Co-alloy particles inducing greater production of both when compared to Ti-alloy particles. These results reiterate that not all metal implant debris induces the same biologic responses despite similarity in dose and size and shape. Thus debris from particular types of implants (modern MoM hip arthroplasty implants) can result in ROS generation that in turn could potentially lead to HIF-1α signaling in THP-1 macrophages.

## Discussion

Collectively, our *in vitro* and *in vivo* data support the contention that metal particulate and soluble degradation products can effect local innate immune responses and tissues in a specific pathophysiologic manner where hypoxic (or hypoxic-like) micro-environment is produced, evidenced by accumulation of HIF-1α protein. High numbers of macrophages in peri-implant tissues are indicative of aseptic loosening and periprosthetic osteolysis [14]. VEGF is integral to this process [14], [15] and is known to be a downstream target of HIF-1α protein production and is a potent angiogenic factor that is up-regulated by macrophages in a hypoxic environment [11]. Until now however, the ability of implant debris to induce HIF-1α reactivity in the context of an innate macrophage response remained unknown. HIF-1α is a potent coping mechanism utilized by macrophages in response to a hypoxic microenvironment used to help mediate the necessary cellular mechanisms in order to maintain activity and viability [8]. Moreover, hypoxia can result in the activation of the NFκB pathway where it has been shown that downstream signaling of TNF-α has a critical role in the biologic reactivity to implants, implant performance and bone loss (inflammatory osteolysis) [20], [21].

Both our *in vitro* and *in vivo* findings indicate Co-alloy is a potent stimuli, eliciting a hypoxic or hypoxic-like microenvironment that can increase the production and stabilization of HIF-1α protein leading to pro-angiogenesis signaling. Importantly this is the first report of a mechanism that can account for the unusual tissue reactions (pseudotumors) that often preferentially accompany high levels of Cobalt metal debris in failed metal-on-metal hip replacements [22]. These data demonstrate that Co-alloy particles induced significant biologic effects, even at low doses (particles per cell), causing macrophages to respond with increases in HIF-1α, TNF-α, VEGF and ROS. In contrast, Ti-alloy particles produced similar effects only at higher challenge doses of particles. Therefore, the hypoxic/hypoxic-like induction of HIF-1α protein in response to metal implant debris may explain how certain kinds of metal-releasing orthopedic implants produce both necrosis/toxicity and neogenic tissue responses resulting in unique mechanisms of poor implant performance.

**Figure 6:**
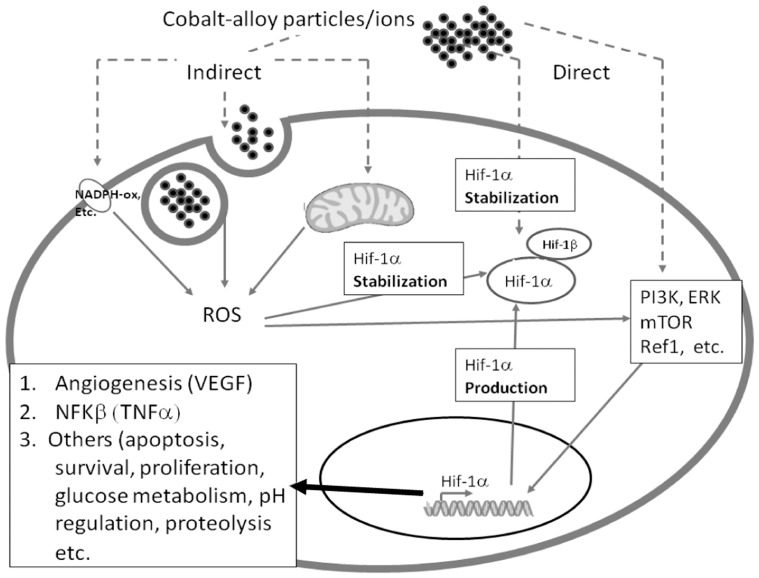
Potential Pathway(s) of HIF-1α Induction to Implant Debris. (**A**) A representation of how metal implant debris (Co-alloy ions and particles) may lead to induction of HIF-1α activity directly or indirectly given our findings.

It is well established that hypoxia can induce inflammation and vise versa, e.g. NFκB[9], that subsequently results in the production of pro-inflammatory cytokines, such as TNF-α. We observed HIF-1α production was enough to induce (or be induced from) NFκB activation by observing increased TNF-α, production a signature cytokine of NFκβ activation. Additionally, the signal transduction pathway of HIF-1α remains elusive but various mechanisms have been proposed linking either increased or decreased ROS generation and either a ROS independent or dependent effect on induction of HIF-1α activity [23]. In order to determine if metals can induce a hypoxic or hypoxic-like state indirectly through known upstream stimulants, we measured ROS production in human THP-1 macrophages. ROS production due to Ti-alloy, Co-alloy particles and Co ion challenge was shown to occur and thus may either cause or contribute to the induction of the HIF-1α associated responses. An important caveat however, is that measurement of ROS by H2DCFDA is prone to auto-oxidation. Therefore, further investigation using additional ROS markers is underway to determine if metal-induced ROS production has a central role in the metal-debris activated of HIF-1α or whether it merely produced simultaneously and if this type of activation can be mitigated by anti-oxidant mediators (**Fig 6**).

In summary, our *in vitro* and *in vivo* data provide evidence that different implant metals induce different levels of hypoxia responses, where Cobalt-alloy soluble and particulate metal debris preferentially induced hypoxia-like pathology resulting in HIF-1α compensatory responses to metal implant debris (vs. Titanium alloy). These *in vitro* and *in vivo* hypoxia-associated responses reported here (e.g. increased HIF-1α, TNF-α, VEGF and ROS) provides a mechanism that can account for the unusual reactions around some implants (e.g. local soft tissue growths “fibro-pseudotumors”) that preferentially release Cobalt particles and ions. Given the increasing number of people receiving total joint replacements (>1 million per year) and the early failures of some designs that release elevated amount of Cobalt [23]–[25], understanding how this occurs is paramount to ensuring the safety and efficacy of current and future designs.
